# A refined wideband acoustical holography based on equivalent source method

**DOI:** 10.1038/srep43458

**Published:** 2017-03-07

**Authors:** Guoli Ping, Zhigang Chu, Zhongming Xu, Linbang Shen

**Affiliations:** 1 The State Key Laboratory of Mechanical Transmission, Chongqing University, Chongqing, 400044, P.R. China; 2 College of Automotive Engineering, Chongqing University, Chongqing, 400044, P.R. China

## Abstract

This paper is concerned with acoustical engineering and mathematical physics problem for the near-field acoustical holography based on equivalent source method (ESM-based NAH). An important mathematical physics problem in ESM-based NAH is to solve the equivalent source strength, which has multiple solving algorithms, such as Tikhonov regularization ESM (TRESM), iterative weighted ESM (IWESM) and steepest descent iteration ESM (SDIESM). To explore a new solving algorithm which can achieve better reconstruction performance in wide frequency band, a refined wideband acoustical holography (RWAH) is proposed. RWAH adopts IWESM below a transition frequency and switches to SDIESM above that transition frequency, and the principal components of input data in RWAH have been truncated. Further, the superiority of RWAH is verified by the comparison of comprehensive performance of TRESM, IWESM, SDIESM and RWAH. Finally, the experiments are conducted, confirming that RWAH can achieve better reconstruction performance in wide frequency band.

Noise source identification based on microphone arrays is commonly used in many acoustical engineering fields. Several techniques such as near-field acoustical holography^1^,^2^,^3^ (NAH) and beamforming^4^,^5^ have been developed to achieve spatial sound field visualization. With good low-frequency resolution and reconstruction performance, NAH has found wide applications in the fields of noise source identification. In past thirty years, NAH has formed a variety of reconstruction algorithms. With poor reconstruction precision, the early NAH based on discrete Fourier transform^1^ (DFT) is limited to the rectangular grid array. The NAH based on boundary element method^6^ (BEM) is not restricted by source shape, but there are complex interpolation and singular integral treatment in BEM. When the statistically optimal near-field acoustical holography^7^ (SONAH) comes out, the algorithm is not limited to the array form and improves the reconstruction precision in the low-medium frequency. Without array form requirement, the Helmholtz equation of the least squares^8^ (HELS) has complex calculation and poor reconstruction precision when the source is non-spherical structure. ESM-based NAH^9^,^10^,^11^ can be adapted to any form of source and array shape. With higher reconstruction precision and computational efficiency, it has been widely studied and applied in recent years.

The basic idea of ESM is that the radiated sound source can be equivalent to the superposition of a series of monopole point sources inside the radiation source. The key of ESM-based NAH is to obtain the equivalent source strength which can be implemented by solving ill-posed morbid equation. To accurately obtain the equivalent source strength, Tikhonov regularization method^12^ is utilized to solve the acoustic inverse problem in traditional ESM-based NAH. In recent years, some scholars have put forward new methods to solve the equivalent source strength. Pereira^13^,^14^,^15^ proposed an iterative weighted ESM (IWESM) based on the traditional Tikhonov regularization ESM (TRESM). Pereira has obtained more accurate equivalent source strength through introducing an iterative weighting matrix based on a spherical array^14^, so as to get better low-frequency reconstruction performance. Bi^16^ applied this algorithm to a planar array, and the low-frequency reconstruction performance was briefly discussed by the simulation. Furthermore, aiming at broadening the upper frequency in acoustical holography, Hald^17^ has presented a steepest descent iteration ESM (SDIESM) based on the ESM model. It is also called wideband acoustical holography^17^ (WAH). The steepest descent method is directly adopted to solve the ill-posed morbid equation and to obtain the equivalent source strength for the SDIESM. The superiority of sound field reconstruction results of the proposed algorithm has been validated by the simulation and experiment of single/dual sources^17^, especially higher reconstruction precision in the medium-high frequency.

Furthermore, acoustical holography is still limited to the Nyquist sampling theorem. To avoid spatial aliasing problems, the array microphone spacing must be somewhat less than half of the acoustic wavelength, which sets a serious limitation on the upper frequency^18^. Hence, the exploration of achieving better reconstruction performance in wide frequency band for acoustical holography is also of great significance. Some scholars have conducted studies. Based on compressive sensing^19^ (CS) technology, the sound field reconstruction performance of NAH has been improved, especially breaking through the upper frequency limitation of the Nyquist sampling theorem. Although Hald has broadened the upper frequency of acoustical holography, regrettably, SDIESM cannot accurately identify multi-source in the low frequency^17^. To solve this issue, it is recommended to adopt TRESM below a transition frequency and switches to SDIESM above that transition frequency^17^. It improves the sound source identification results of multi-source in the low frequency, but the effect is still not satisfactory. This paper is motivated to address this issue. A novel refined wideband acoustical holography (RWAH) is proposed on the basis of IWESM and SDIESM. RWAH can further enhance the performance of sound field reconstruction and sound source identification in wide frequency band.

## Methods

### RWAH

The basic principle of ESM-based NAH is solving the equivalent source strength. And these equivalent sources strength can be obtained by the measured sound pressure on the holographic plane. Input data for ESM-based NAH can be obtained by simultaneous acquisition with an array of *M* microphones, followed by averaging of the *M* × *M* element cross-power spectral matrix **A**_(*M*×*M*)_, whose eigenvalue decomposition (EVD) is written as:


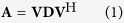


where **V** is a unitary matrix with the columns containing the eigenvectors **v**_*μ*_, *μ* = 1, 2, …, *M*; **D** is a diagonal matrix with the eigenvalues *d*_*μ*_ on the diagonal. The *μ*th order principal component **p**_*μ*_ is calculated as:


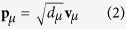


Each of these principal components is independently applied to the ESM-based NAH, and the output is added on a power basis. Now considering only a certain principal component **p**, so we overlook the index *μ*. That is to say, the input data is a single vector **p** with measured sound pressure for all microphones. Assuming that there are _N_ equivalent sources on source plane, the input data can be expressed as the following matrix form:


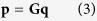


where **q** is the equivalent source strength; **G** is the transfer matrix between the measured sound pressure and equivalent sources, and **G**(*m, n*) is the transfer function between the *m*th microphone and the *n*th equivalent source, which is expressed as:


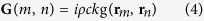


where *ρ* is air density; *c* is sound velocity; *k* is wave-number; **r**_*m*_ is the vector of the *m*th microphone position; **r**_*n*_ is the vector of the *n*th equivalent source position; g(**r**_*m*_, **r**_*n*_) is the Green function, whose expression is as follow:


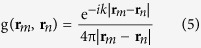


In practice, a large number of equivalent sources are arranged to obtain the detail information of sources, i.e. *N* > *M*. This results in an ill-posed acoustical inverse problem and under-determined equation solving in equation (3), so the equivalent source strength has no particular solution.

Fortunately, there are multiple solving methods for the equation (3), such as TRESM^11^, IWESM^14^ and SDIESM^17^. To achieve better reconstruction performance in wide frequency band, a refined wideband acoustical holography (RWAH) is proposed. RWAH adopts IWESM below a transition frequency and switches to SDIESM above that transition frequency. Besides, the principal components of cross-power spectral matrix of measured sound pressure in RWAH have been truncated. The transition frequency *f*_*t*_ is defined as^17^:


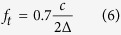


where ∆ is the average spacing between microphones. This paper adopts Brüel&Kjær 36-channel Combo array with the microphone spacing of 0.1 m, so the transition frequency is about 1200 Hz. The basic principles of principal components truncation (PCT), IWESM and SDIESM in RWAH are respectively introduced as follows.

### PCT

The cross-power spectral matrix of measured sound pressure is firstly decomposed by EVD to get each order principal component, and then all the principal components are traversed for the reconstruction calculation. In fact, the reconstruction sound pressure is mainly determined by the principal components of larger eigenvalues, which are corresponding to the real sources; while the principal components of smaller eigenvalues are corresponding to the ghost sources. Therefore, in order to improve the computational efficiency and possibly to eliminate noise interference, the principal components can be truncated. Assuming that retaining *l* order principal components, the *l*th order principal component **p**_*l*_ can be expressed as:


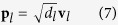


where *d*_*l*_ is the *l*th eigenvalue, and **v**_*l*_ is the corresponding eigenvector. To achieve the reasonable truncation of eigenvalue, this paper introduces a truncation threshold 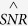, and defines the truncated condition as follow:


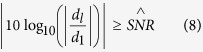


In this paper, 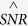 is taken as 20 dB to remove the source whose strength is weaker than the main source more than 20 dB.

### IWESM

The basic idea of TRESM is utilizing Tikhonov regularization technology^12^ to solve the equivalent source strength by minimizing the following function:


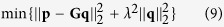


where *λ* is the regularization parameter, which can be determined by L-curve method^20^, GCV method^21^ and Bayesian regularization criterion method^22^,^23^. The basic idea of IWESM is introducing a weighting matrix **W** to the norm solution and converting the constraint term 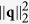 into 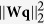 in the equation (9). So the minimization problem of equation (9) can be expressed as:


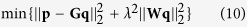


where the **W** is a invertible diagonal matrix with non-zero diagonal entries, which meets for **WW**^−1^ = **I**. And 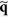 is defined as 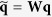, which is substituted into the equation (10):


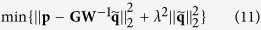


The equation (11) is promptly recognized as the standard-form of Tikhonov regularization, whose solution can be written as:


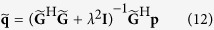


where a new transfer matrix is defined as 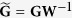, whose SVD is defined by:


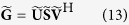


Substituting the equation (13) into the equation (12):


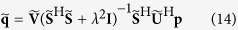


After the above weighting treatment, the equivalent source strength of equation (3) can be given by:


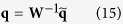


The key step of the above solving is to construct the weighting matrix **W**, which is implemented by an iterative manner^15^. The equations (10),(11),(12),(13),(14),(15), need repeatedly calculating before a new weighting matrix comes out.

### SDIESM

The basic idea of SDIESM is iteratively solving the equation (3) by utilizing the steepest descent method, and the process is introduced as follows: defining the residual vector 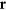 and the quadratic residual function *F*,


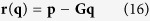



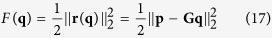


First, the *i*th iteration step ∆**q**^(*i*)^ is calculated by minimizing the residual function *F* in the steepest descent direction:


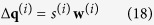


where **w**^(*i*)^ is the negative gradient vector:


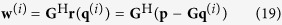


*s*^(*i*)^ is the step length to the minimum along that direction:


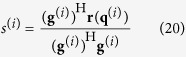


where the vector **g**^(*i*)^ is defined as:


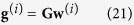


The iteration step is obtained from the equations (16),(17),(18),(19),(20),(21), so the iterative equation of equivalent source strength is given by:


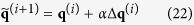


where *α* is a possible relaxation factor, typically between 0.5 and 1.0.

However, just using the equation (22) for sound field reconstruction will lead to the introduction of ghost sources which are associated with the real sources and weaker than the strongest real source. To reduce their influence on the real sources, we can suppress and eliminate ghost sources by setting all components in 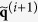 below a certain threshold to zero. The value of this threshold is recommended as^17^:


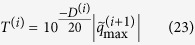


where *D*^(*i*)^ is the dynamic range of retained source amplitudes, dB; 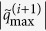 is the amplitude of largest element in 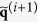. After the above truncation treatment, the equivalent source strength of (*i* + 1) th iteration can be expressed as:


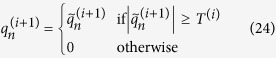


Meanwhile, *D*^(*i*)^ is updated during the iteration in the following way:


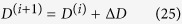


Finally, the steepest descent algorithm will be terminated when it meets the condition in the equation (26).


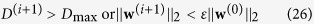


where *D*_max_ is an upper limit on *D*^(*i*)^ and *ε* is a small number. The following initial values are given by^17^:


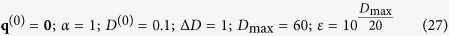


The above is the basic principle of RWAH, which includes the PCT, IWESM and SDIESM. To intuitively describe the RWAH algorithm, its theoretical flow chart is presented in Supplementary Information.

### Simulation conditions

A monopole point source and two coherent in-phase monopole point sources were taken as examples for simulation analysis. Simulation conditions are as follows: the point source with unit strength is simulated at 200–6000 Hz frequency band with a step of 100 Hz. The single source is located at (0, 0, 0) m and the dual sources are located at (0.14 m, 0, 0) and (−0.14 m, 0, 0). Holographic plane is the Brüel&Kjær 36-channel Combo array with the diameter of 0.65 m and the holographic distance is 0.1–0.3 m. The distance between the sources and the reconstruction plane is 0.05 m. The number of equivalent sources is 31 × 31, which are arranged evenly with the spacing of 0.02 m. White Gaussian noise is added to the measured sound pressure with the SNR of 40 dB. Bayesian regularization criterion^22^,^23^ method is chosen to determine the regularization parameter in the TRESM and IWESM algorithms. The relative error (RE) is given to evaluate the reconstruction performance, whose expression^6^ is defined as:


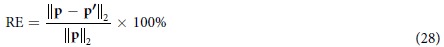


where **p**′ and **p** are the sound pressure values of the reconstruction and theory results, respectively.

### Data measurement

Experiments were conducted in a semi-anechoic chamber. Sound pressure signals of loudspeakers were measured by the Brüel&Kjær 36-channel Combo array at 0.2 m distance from loudspeakers. The array utilizes Brüel&Kjær Type 4951 microphones and has a diameter of 0.65 m^11^. Sound pressure signals received by microphones are acquired simultaneously by Brüel&Kjær 41-channel PULSE Type 3560D Data Acquisition System and then transferred to PULSE LABSHOP Software where their cross-spectra are achieved^11^. Finally, TRESM, IWESM, SDIESM and RWAH are applied to reconstruct the sound field at 0.05 m distance from loudspeakers by MATLAB programming. Beyond the array measurement, the theory result of reconstruction plane was measured by Brüel&Kjær Type 4189 microphone. A scan of 8 × 8 positions with 0.05 m spacing was performed by microphone.

## Results

**Figure 1:**
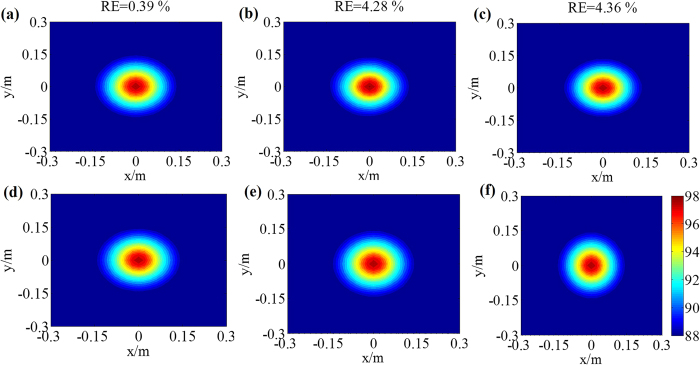
Reconstruction results of RWAH for single source. **(a,b** and **c)** are the reconstruction results of RWAH at the frequency of 500 Hz, 2000 Hz and 4000 Hz, respectively. **(d,e** and **f)** are the theory sound pressure level (unit: dB) of the frequency of 500 Hz, 2000 Hz and 4000 Hz, respectively.

**Figure 2:**
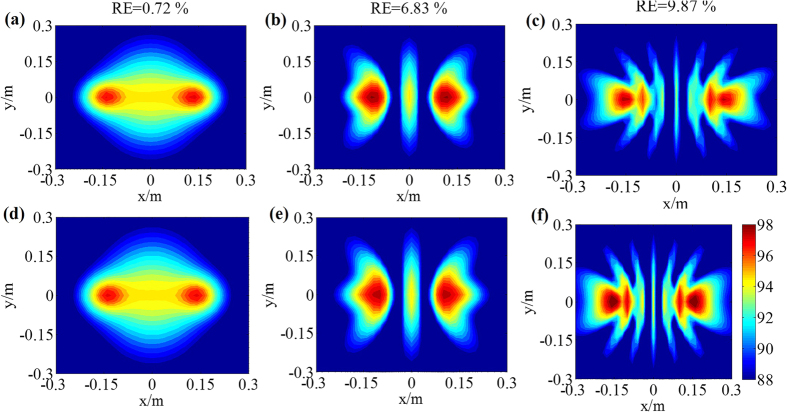
Reconstruction results of RWAH for dual sources. **(a,b** and **c)** are the reconstruction results of RWAH at the frequency of 500 Hz, 2000 Hz and 4000 Hz, respectively. **(d**,**e** and **f)** are the theory sound pressure level (unit: dB) of the frequency of 500 Hz, 2000 Hz and 4000 Hz, respectively.

To analyze the performance of sound field reconstruction and sound source identification of the proposed RWAH, the reconstruction results of RWAH were presented under the single/dual sources of 500 Hz, 2000 Hz and 4000 Hz. The contour maps of each figure use the same colorbar. Figures 1 and 2 show that, when the sources frequency is 500 Hz, 2000 Hz and 4000 Hz, the reconstruction result and theory result of RWAH are basically consistent. The relative errors are all less than 10%, particularly worth mentioning is that RWAH can accurately identify the dual sources of compact distribution at 500 Hz. In addition, for the single principal component of input data, the calculating time of RWAH is 6.867 s below the transition frequency and is 3.888 s above the transition frequency. Obviously, the proposed RWAH has good resolution and computational efficiency, and the reconstruction precision is high. In fact, even in the case of the multiple sources, the conclusions are consistent.

## Discussion

**Figure 3:**
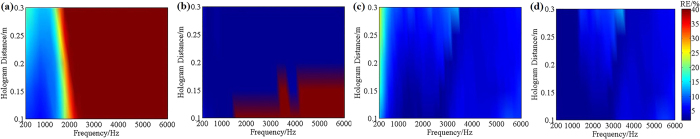
Relative errors of four algorithms under the frequencies of 200–6000 Hz and the holographic distances of 0.1–0.3 m for single source. **(a)** TRESM. **(b)** IWESM. **(c)** SDIESM. **(d)** RWAH.

**Figure 4:**
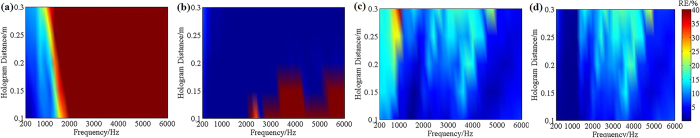
Relative errors of four algorithms under the frequencies of 200–6000 Hz and the holographic distances of 0.1–0.3 m for dual sources. **(a)** TRESM. **(b)** IWESM. **(c)** SDIESM. **(d)** RWAH.

To validate the superiority of the proposed RWAH, the comprehensive performance of TRESM, IWESM, SDIESM and RWAH was discussed in this section. Based on the above simulation conditions, the relative error contour maps of reconstruction results of four algorithms are presented in Figs 3 and 4 under the frequencies of 200–6000 Hz and holographic distances of 0.1–0.3 m. The contour maps adopt the same colorbar with the upper limit of 40%.

Figures 3 and 4 show that, the relative error of TRESM is larger in the medium-high frequency, especially larger than 40% above 2000 Hz. For the IWESM, the relative error is extremely small below 1800 Hz, which is less than 5%. While above 1800 Hz and the holographic distances are 0.1 m, 0.15 m and 0.2 m, the relative error is much exceeding 40%; the existing over-regularization is due to the larger weighting matrix **W** at this time, which is an inherent defect in IWESM. For the SDIESM, the overall reconstruction precision is higher than TRESM and IWESM. The relative error is extremely small when the frequency is above 1000 Hz; while the reconstruction precision is slightly low below 1000 Hz, which is inferior to IWESM. For the RWAH, the relative error is nicely small in wide frequency band for single/dual sources. Compared to TRESM, IWESM and SDIESM, the reconstruction precision of RWAH is the highest in the entire frequency band at different holographic distances.

To sum up, the advantages and disadvantages of TRESM, IWESM, SDIESM and RWAH algorithms can be identified by analyzing their reconstruction precision in wide frequency band. The results are shown in Table 1, where the more “★” indicates the better performance and the “☆” indicates not applicable. Table 1 shows that the proposed RWAH has better performance of sound field reconstruction and sound source identification in wide frequency band.

**Figure 5:**
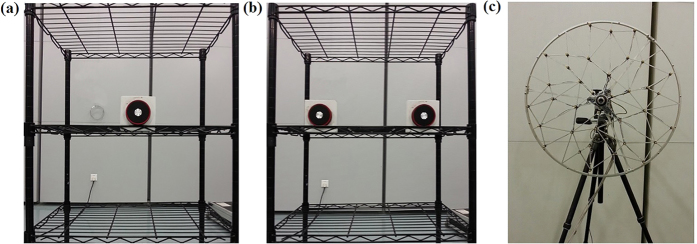
Experimental site. **(a)** Single source, **(b)** Dual sources, **(c)** Combo array.

**Figure 6:**
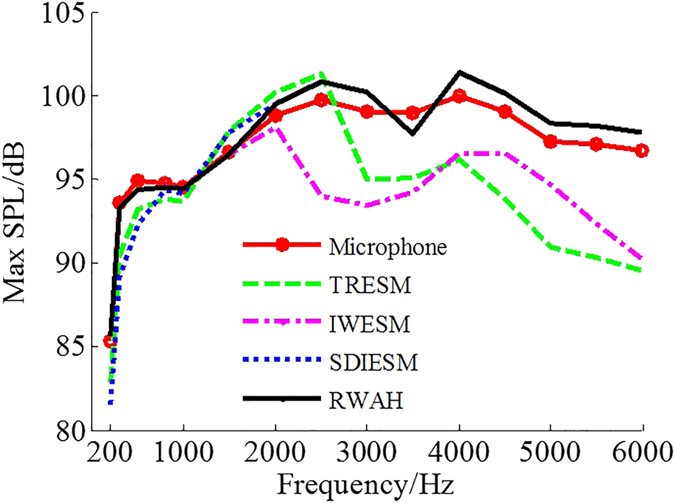
Maximum SPL of the microphone measurement and reconstruction results of four algorithms.

**Figure 7:**
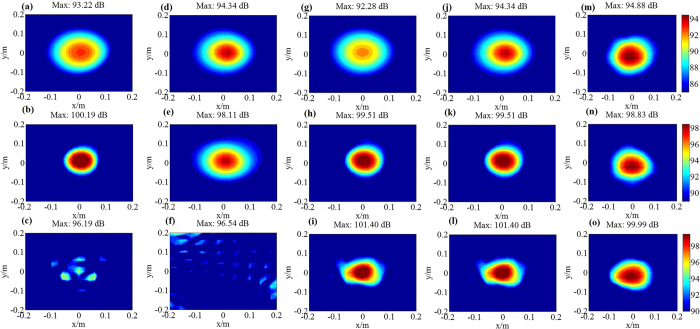
Experiment results of TRESM, IWESM, SDIESM and RWAH algorithms for single source. **(a,b** and **c)** are the reconstruction results of TRESM at the frequency of 500 Hz, 2000 Hz and 4000 Hz, respectively. **(d,e** and **f)** are the reconstruction results of IWESM at the frequency of 500 Hz, 2000 Hz and 4000 Hz, respectively. **(g,h** and **i)** are the reconstruction results of SDIESM at the frequency of 500 Hz, 2000 Hz and 4000 Hz, respectively. **(j,k** and **l)** are the reconstruction results of RWAH at the frequency of 500 Hz, 2000 Hz and 4000 Hz, respectively. **(m,n** and **o)** are the microphone measurement sound pressure level (unit: dB) of the frequency of 500 Hz, 2000 Hz and 4000 Hz, respectively.

**Figure 8:**
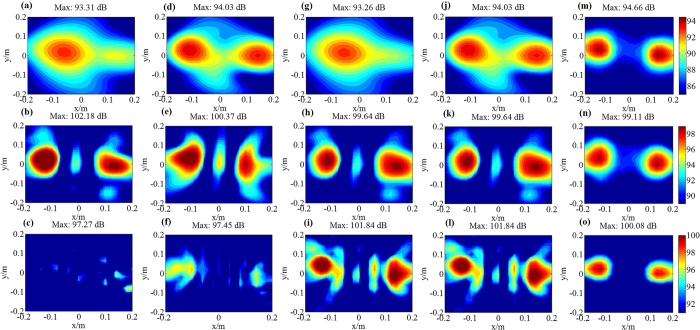
Experiment results of TRESM, IWESM, SDIESM and RWAH algorithms for dual sources. **(a,b** and **c)** are the reconstruction results of TRESM at the frequency of 500 Hz, 2000 Hz and 4000 Hz, respectively. **(d,e** and **f)** are the reconstruction results of IWESM at the frequency of 500 Hz, 2000 Hz and 4000 Hz, respectively. **(g,h** and **i)** are the reconstruction results of SDIESM at the frequency of 500 Hz, 2000 Hz and 4000 Hz, respectively. **(j,k** and **l)** are the reconstruction results of RWAH at the frequency of 500 Hz, 2000 Hz and 4000 Hz, respectively. **(m,n** and **o)** are the microphone measurement sound pressure level (unit: dB) of the frequency of 500 Hz, 2000 Hz and 4000 Hz, respectively.

To further validate the superiority of RWAH, the experiments were conducted. Figure 5 shows the experimental site, where the single source is located in (0, 0, 0) and the dual sources are located in (−0.14 m, 0.03 m, 0) and (0.14 m, 0, 0). When the single source is 200–6000 Hz, the maximum sound pressure level (SPL) of the microphone measurement results and the reconstruction results of four algorithms is shown in Fig. 6. In addition, when the single/dual sources are 500 Hz, 2000 Hz and 4000 Hz, the microphone measurement SPL and the reconstruction SPL of TRESM, IWESM, SDIESM and RWAH are presented in Figs 7 and 8. The contour maps of each frequency use the same colorbar.

Figures 7 and 8 show that TRESM and IWESM are unable to identify the single/dual sources at 4000 Hz. At the frequency of 500 Hz, the difference between the reconstruction results of SDIESM and the microphone measurement results is slightly larger in Fig. 7. In Fig. 8, SDIESM cannot identify the two sources, whose reconstruction result is only one source of mutual fusion. While RWAH can accurately identify the dual sources of compact distribution at 500 Hz. The reconstruction results of RWAH and the microphone measurement results are most consistent under single/dual sources at the frequency of 500 Hz, 2000 Hz and 4000 Hz, which can accurately achieve the sources positioning and truthfully reflect the sound pressure values. In addition, from the Fig. 6, conclusion can be drawn that the gaps of maximum SPL between the reconstruction results of RWAH and the microphone measurement results are always the smallest among four algorithms in the frequencies of 200–6000 Hz. That is to say, the reconstruction results of RWAH are closest to the measurement results in wide frequency band. The experimental results have validated the correctness that RWAH can achieve better reconstruction performance in wide frequency band.

To achieve better reconstruction performance in wide frequency band, a refined wideband acoustical holography (RWAH) is proposed. RWAH adopts IWESM below a transition frequency and switches to SDIESM above that transition frequency, and the principal components of input data in RWAH have been truncated. In conclusion, RWAH not only inherits the respective advantages of IWESM and SDIESM, but also avoids their deficiency, and the computational efficiency is improved by principal components truncation. On the basis of IWESM, the reconstruction performance of medium-high frequency is enhanced. On the basis of SDIESM, the low-frequency reconstruction precision and resolution are all improved. RWAH can further enhance the performance of sound field reconstruction and sound source identification in wide frequency band.

## Additional Information

**How to cite this article**: Ping, G. *et al*. A refined wideband acoustical holography based on equivalent source method. *Sci. Rep.* **7**, 43458; doi: 10.1038/srep43458 (2017).

**Publisher's note:** Springer Nature remains neutral with regard to jurisdictional claims in published maps and institutional affiliations.

## Supplementary Material

Supplementary Information

## Figures and Tables

**Table 1:** Advantages and disadvantages of TRESM, IWESM, SDIESM and RWAH.

Algorithms	Low-frequency precision	Medium-frequency precision	High-frequency precision	Holographic distance adaptability	Computational efficiency
TRESM	★★	★	☆	★★	★★★
IWESM	★★★★	★★	☆	★	★★
SDIESM	★	★★★★	★★★★	★★★	★
RWAH	★★★★	★★★★	★★★★	★★★	★★
