# Supplementary material for: A refined wideband acoustical holography based on equivalent source method
Source: Sci Rep. 2017 Mar 7;7:43458. doi: 10.1038/srep43458 (PMC5339900; doi:10.1038/srep43458)
Supplement: Supplementary Information [file srep43458-s1.pdf]

A refined wideband acoustical holography based on equivalent source method

Guoli Ping[1, 2], Zhigang Chu[1, 2, *], Zhongming Xu[1] and Linbang Shen[1]

Supplementary Information

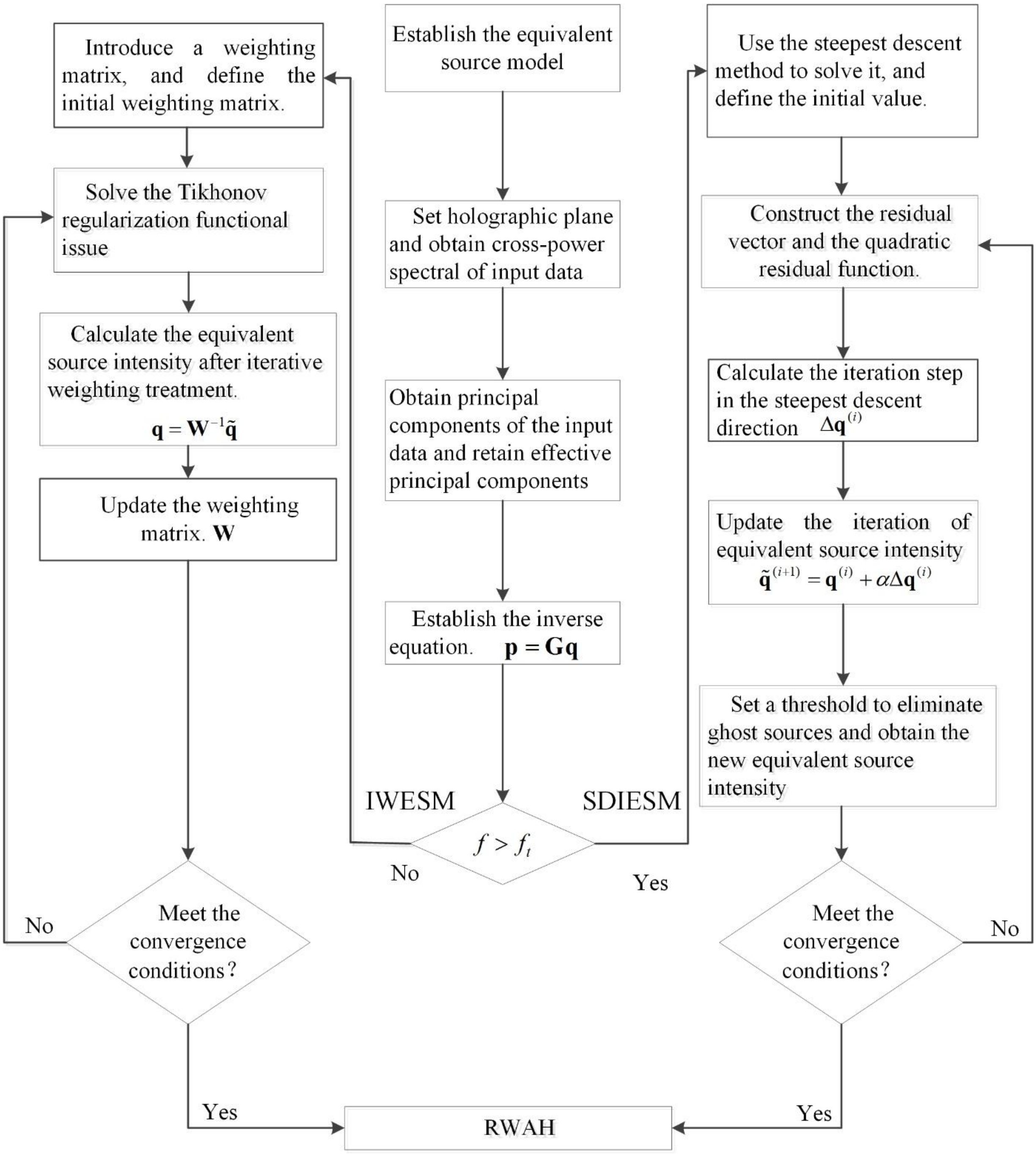

Figure 1S. Theoretical flow chart of RWAH.
